# PCR-Based Assays versus Direct Sequencing for Evaluating the Effect of KRAS Status on Anti-EGFR Treatment Response in Colorectal Cancer Patients: A Systematic Review and Meta-Analysis

**DOI:** 10.1371/journal.pone.0107926

**Published:** 2014-09-26

**Authors:** Lianfeng Shan, Ming Li, Jianzhong Ma, Huidan Zhang

**Affiliations:** 1 Department of Mathematics, College of Basic Medical Sciences, China Medical University, Shenyang, China; 2 Department of Cell Biology, Key Laboratory of Cell Biology, Ministry of Public Health, and Key Laboratory of Medical Cell Biology, Ministry of Education, China Medical University, Shenyang, China; H. Lee Moffitt Cancer Center & Research Institute, United States of America

## Abstract

**Background:**

The survival rate of colorectal cancer (CRC) patients carrying wild-type KRAS is significantly increased by combining anti-EGFR monoclonal antibody (mAb) with standard chemotherapy. However, conflicting data exist in both the wild-type KRAS and mutant KRAS groups, which strongly challenge CRC anti-EGFR treatment. Here we conducted a meta-analysis in an effort to provide more reliable information regarding anti-EGFR treatment in CRC patients.

**Methods:**

We searched full reports of randomized clinical trials using Medline, the American Society of Clinical Oncology (ASCO), and the European Society for Medical Oncology (ESMO). Two investigators independently screened the published literature according to our inclusive and exclusive criteria and the relative data were extracted. We used Review Manager 5.2 software to analyze the data.

**Results:**

The addition of anti-EGFR mAb to standard chemotherapy significantly improved both progression-free survival (PFS) and median overall survival (mOS) in the wild-type KRAS group; hazard ratios (HRs) for PFS and mOS were 0.70 [95% confidence interval (CI), 0.58–0.84] and 0.83 [95% CI, 0.75–0.91], respectively. In sub-analyses of the wild-type KRAS group, when PCR-based assays are employed, PFS and mOS notably increase: the HRs were 0.74 [95% CI, 0.62–0.88] and 0.87 [95% CI, 0.78–0.96], respectively. In sub-analyses of the mutant KRAS group, neither PCR-based assays nor direct sequencing enhance PFS or mOS.

**Conclusion:**

Our data suggest that PCR-based assays with high sensitivity and specificity allow accurate identification of patients with wild-type KRAS and thus increase PFS and mOS. Furthermore, such assays liberate patients with mutant KRAS from unnecessary drug side effects, and provide them an opportunity to receive appropriate treatment. Thus, establishing a precise standard reference test will substantially optimize CRC-targeted therapies.

## Introduction

Over the last two decades, considerable progress regarding the molecular biology of colorectal cancer (CRC) has remarkably increased the biologic therapeutic options [1]. A key breakthrough was the discovery of two monoclonal antibodies (mAb) targeting epidermal growth factor receptor (EGFR): chimeric immunoglobulin G1 mAb (cetuximab) and a fully humanized immunoglobulin G2 mAb (panitumumab). These antibodies have been found to be very effective in combination with standard chemotherapy or as single therapeutic agents for chemotherapy-resistant metastatic CRC (mCRC) [2], [3]. In 2004, the United States Food and Drug Administration (FDA) approved cetuximab as the first mAb inhibiting EGFR for the treatment of mCRC, which was followed by approval of panitumumab in 2006 [4], [5]. Unfortunately, nearly one third of mCRC patients do not benefit from this targeted therapy but also experience consequential side effects [6], [7]. Thus, it is crucial to identify those patients who are most likely to respond to achieve personalized treatment. KRAS protein is a key signaling molecule between extracellular EGFR ligands and signaling in cells. Extensive retrospective studies and phase III trials disclosed that KRAS gene activating mutations are the main negative predictor of mCRC anti-EGFR therapy [8]–[10]. Based on these findings, the FDA changed the guidelines to recommend that cetuximab and panitumumab only be given to CRC patients with wild-type KRAS [11]. However, researchers continue reporting conflicting facts in both the KRAS wild-type and mutant groups: for example, patients carrying wild-type KRAS do not respond, whereas those carrying mutant KRAS did [12]–[15]. Such contradictory data strongly challenge mCRC treatment. Regardless of the sporadically reported contribution of other gene variations, such as BRAF mutations, PIK3CA mutations, and loss of PTEN expression [16]–[19], the accuracy of genotyping methods might explain this phenomenon. For example, one experimental study supports this hypothesis by showing highly sensitive methods for detection of KRAS mutations identified 13 additional mCRC patients resistant to anti-EGFR mAb compared with direct sequencing [20].

To systematically address this issue, we conducted a systematic review and meta-analysis to assess progression-free survival (PFS) and median overall survival (mOS) in patients whose KRAS status were detected by either PCR-based assays or direct sequencing. We compared the ability of these two genotyping methods to evaluate the effect of KRAS status on response to CRC anti-EGFR treatment.

## Methods

### Search strategy

**Figure 1:**
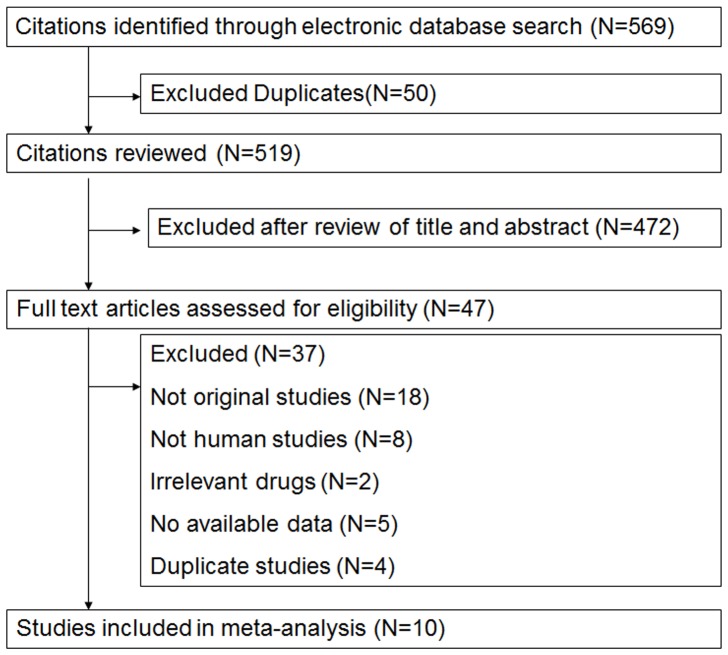
Flow chart of the trial selection.

The deadline for trial publication was December 31, 2013. Full reports of randomized clinical trials that addressed the effect of KRAS status on response to CRC anti-EGFR treatment were gathered through Medline (PubMed: www.ncbi.nlm.nih.gov/PubMed), the American Society of Clinical Oncology (ASCO, www.asco.org), and the European Society for Medical Oncology (ESMO, www.esmo.org). The keywords used for searching were: CRC, KRAS mutation, cetuximab, panitumumab, chemotherapy, randomized, and anti-EGFR mAb. We first excluded double antibody protocols that also evaluated vascular endothelial growth factor (VEGF) antibody. We then searched the target trials according to the workflow shown in Figure 1.

### Patient groups and subgroups

To evaluate the overall effect of anti-EGFR mAb drugs as an addition to standard chemotherapy, we divided all enrolled patients into two groups: the experimental group, treated with a combination of anti-EGFR mAb and standard chemotherapy; and the control group, treated with standard chemotherapy only. We then analyzed the effect of KRAS status on response to anti-EGFR treatment by further dividing patients into the wild-type KRAS and mutant KRAS groups. To perform sub-analyses to compare the ability of different genotyping methods to evaluate the effect of KRAS status on response to CRC anti-EGFR treatment, we separated patients with different KRAS status into two subgroups: the PCR-based assay subgroup and the direct sequencing subgroup.

### Statistical analyses

We extracted hazard ratios (HRs) for PFS and mOS with 95% confidence intervals (CIs) from the enrolled trials. PFS and mOS data for each trial are represented by the log of the HR and its variance of experimental compared with the control group in both patients with wild-type and mutant KRAS, according to a previously described method [21]. A HR <1 indicates an improvement in PFS or mOS. If the trials reported log HR and variance, we used them directly. If the trials did not provide these values, we extracted the data from survival curves, when available, to estimate the values of the log HR and variance. When survival curves for the treatment groups were not available, other data, such as log-rank test P values and the number of events in each group, were extracted to allow estimation of the log HR and variance. To obtain summary HRs of the experimental group compared with the control group, the log HRs were pooled using a random-effect model or fixed-effect model for continuous outcomes with CIs set at 95% significance [22]. To determine the appropriate model, we employed Review Manager 5.2 software to analyze inter-study heterogeneity. When the *P* value for heterogeneity was less than 0.05, we chose a random-effect model. When the *P* value for heterogeneity was greater than or equal to 0.05, we chose a fixed-effect model.

## Results

### Selected Trials

We identified ten trials according to the workflow shown in Figure 1 [13], [23]–[31]. These trials are listed in Table 1. A total of 6699 patients were evaluated in this meta-analysis.

**Table 1:** Characteristics of trials enrolled in the meta-analysis.

Study	Wild-type		Mutant		Detection Method	Major Findings	
	Anti-EGFR	Control	Anti-EGFR	Control			
Karapetis 2008	110	105	75	75	Direct sequencing	PFS	OS
Langer 2008	86	86	54	54	Direct sequencing	PFS	-
Amado 2008	124	119	84	100	PCR assay	PFS	OS
Bokemayer 2009	61	73	52	47	PCR assay	PFS	-
Van Cutsem 2009	172	176	105	87	PCR assay	PFS	OS
Douillard 2010	325	331	221	219	PCR assay	PFS	OS
Maughan 2010	362	367	281	281	PCR assay	PFS	-
Peeters 2010	303	294	238	248	PCR assay	PFS	OS
Kjell 2012	97	97	72	58	PCR assay	PFS	OS
Douillard 2013	259	253	272	276	PCR assay	PFS	OS

### Progression-Free Survival (PFS)

**Figure 2:**
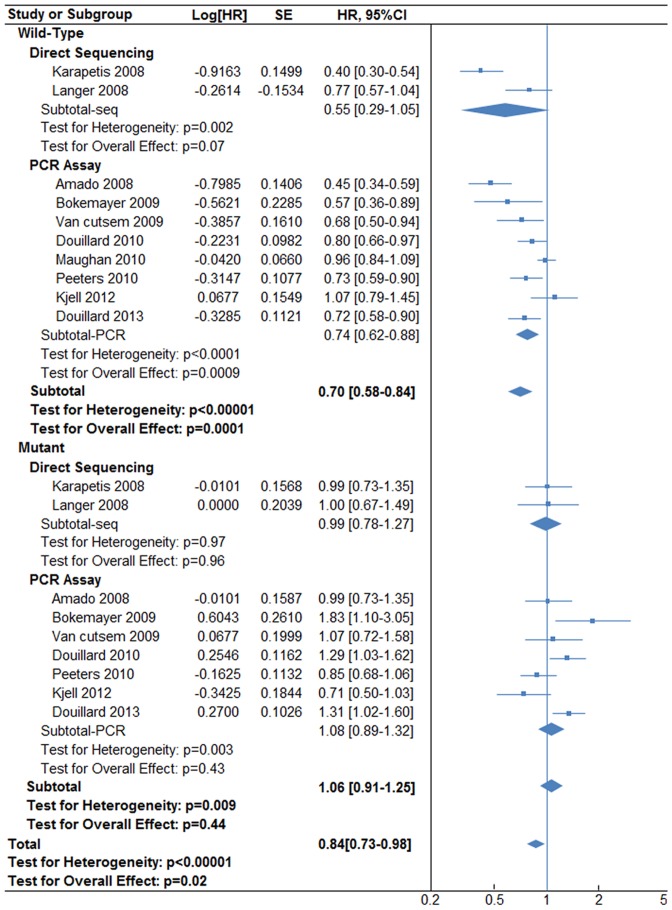
Progression-free survival (PFS) associated with anti-EGFR plus chemotherapy versus chemotherapy alone, according to KRAS status and genotyping methods.

The results showing the addition of anti-EGFR therapy to standard chemotherapy are presented in Figure 2. For all patients, with or without KRAS mutations, addition of anti-EGFR therapy remarkably improved PFS [HR 0.84, (95% CI, 0.73–0.98), P = 0.02] according to a random-effect model (P value for heterogeneity <0.00001). When we evaluated the data based on KRAS status, we observed a significant improvement in PFS in patients with wild-type status [HR 0.70, (95% CI, 0.58–0.84), P = 0.0001] according to a random-effect model (P value for heterogeneity <0.00001), but not in patients with mutant KRAS [HR 1.06, (95% CI, 0.91–1.25), P = 0.44] according to a random-effect model (P value for heterogeneity  = 0.009). In sub-analyses of the wild-type KRAS group, PFS considerably increased in the PCR-based assay subgroup [HR 0.74, (95% CI, 0.62–0.88), p = 0.0009] according to a random-effect model (P value for heterogeneity <0.0001), but not in the direct sequencing subgroup [HR 0.55, (95% CI, 0.29–1.05), P = 0.07] according to a random-effect model (P value for heterogeneity  = 0.002). In sub-analyses of the mutant KRAS group, neither PCR-based assay nor direct sequencing enhanced PFS [HR 0.99, (95% CI, 0.78–1.27), p = 0.96], according to a fixed-effect model (P value for heterogeneity  = 0.97) and [HR 1.08, (95% CI, 0.89–1.32), p = 0.43] according to a random-effect model (P value for heterogeneity = 0.0003), respectively.

### Median Overall Survival (mOS)

**Figure 3:**
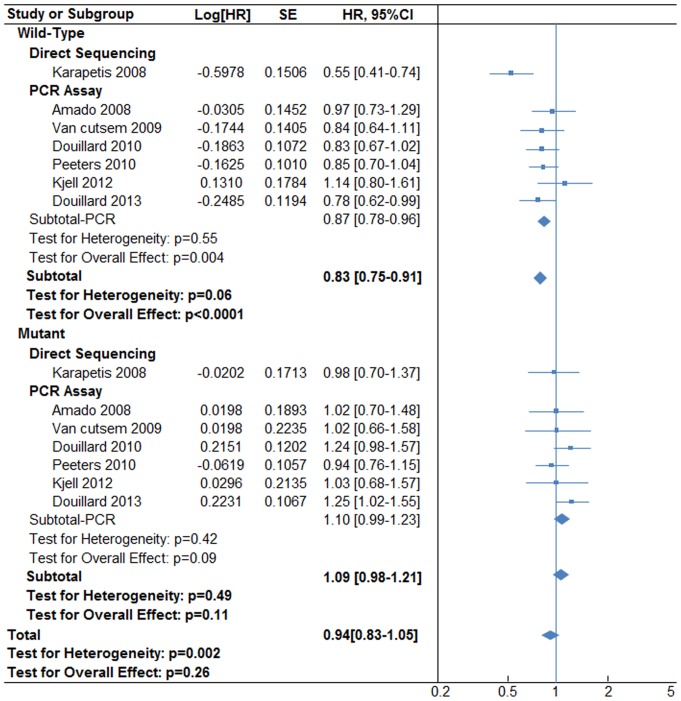
Median overall survivals (mOS) associated with anti-EGFR plus chemotherapy versus chemotherapy alone, according to KRAS status and genotyping methods.

For overall patients, addition of anti-EGFR does not remarkably improve the mOS [HR 0.94, (95% CI, 0.83–1.05), p = 0.26] according to a random-effect model (P value for heterogeneity = 0.002). However, we observed significant enhancement in mOS in the wild-type KRAS group [HR 0.83, (95% CI, 0.75–0.91), P<0.0001] according to a fixed-effect model (P value for heterogeneity  = 0.06). In sub-analyses of the wild-type KRAS group, mOS increased in the PCR-based assay subgroup, [HR 0.87, (95% CI, 0.78–0.96), P = 0.004] according to a fixed-effect model (P value for heterogeneity  = 0.55). The direct sequencing subgroup only included one trial and is not applicable for meta-analysis. In the mutant KRAS group, there was no obvious improvement in mOS [HR 1.09, (95% CI, 0.98–1.21), P = 0.11] according to a fixed-effect model (P value for heterogeneity  = 0.49). In sub-analyses of the mutant-type KRAS subgroup, PCR-based assays did not obviously change the therapeutics for CRC [HR 1.10, (95% CI, 0.99–1.23), P = 0.009] according to a fixed-effect model (P value for heterogeneity  = 0.42). In the direct sequencing subgroup, the single trial does not allow for meta-analysis. These data are shown in Figure 3.

## Discussion

New molecular-targeted therapies have been widely used in CRC treatment with the distinguished advantages of high specificity and low toxicity [32]. Current evidence has approved that CRC patients with wild-type KRAS will respond to anti-EGFR treatment. However, some patients with wild-type KRAS do not exhibit the expected response, whereas some patients with mutant KRAS do respond well. A major explanation for such contradictory observation might be because the accuracy of current genotyping methods varied among different labs and different clinical trials. In this study, we systematically analyze the capabilities of PCR-based assays versus direct sequencing to evaluate the effect of KRAS status on response to anti-EGFR treatment.

As expected, the present meta-analysis confirms the clinical benefits when anti-EGFR therapy is added to standard chemotherapy for the treatment of mCRC patients harboring wild-type KRAS, with a significant improvement in PFS. When we performed sub-analyses based on the different genotyping methods, both PFS and mOS improved when PCR-based assays were employed. In these 10 trials, researchers used PCR-based assays, including the PCR clamping-melting curve method and allele-specific real-time PCR, to detect KRAS mutations. The sensitivities of these two methods are 0.1% and 0.5% [33], [34], respectively. In comparison, the sensitivity of direct sequencing is 10–20% [35]. Thus, PCR-based methods enable sensitive detection of low abundance KRAS mutations, increase the detection rate, exclude most patients with mutant KRAS, and therefore improve PFS and mOS. Thus, we have exposed a new clue regarding the contradiction in mCRC treatment: there is a subpopulation of mCRC patients carrying low-level KRAS mutations between patients with wild-type KRAS and those with abundant mutant KRAS. Due to limited sensitivity of the analysis methods, this subpopulation of patients is typically not grouped correctly, causing the treatment to go in the wrong direction. This results in an unpredictable outcome for individual patients, as well as dramatically confuses the mCRC treatment strategy. A solution for this issue will significantly improve treatment of mCRC patients.

Compared to the high specificity of direct sequencing, we must consider the specificity of PCR-based assays when we use genotyping results to estimate the efficiency of anti-EGFR targeted therapy. The potentially high false-positive error rate of PCR-based assays might exclude patients carrying wild-type KRAS from receiving the correct treatment. Here we propose that PCR-based assays will group some wild-type KRAS patients into the mutant group, and thus incorrectly increase PFS and mOS of the mutant group. Interestingly, we did not observe this phenomenon in our study, which suggests that the PCR-based assays used in these trials are specific enough to disclose mutant KRAS. One of these assays is PCR clamping and the melting curve method. The clamp used here is peptide nucleic acid (PNA) in which the sugar phosphate backbone of natural nucleic acid is replaced by a synthetic peptide backbone and recognizes a sequence specific DNA obeying the Watson-Crick hydrogen bonding scheme [36]. The PNA-DNA heteroduplex exhibits extraordinary thermal stability, whereas incorporation of any mismatch in the duplex will lower the thermal stability by more than 10°C; this enables highly specific mutation detection [33], [36]. Mutation detection by allele-specific real-time PCR is based on the principle that extension is efficient when the 3′ terminal base of a primer matches its target, whereas extension is inefficient or nonexistent when the terminal base is mismatched, and also showed good specificity in previous applications [37], [38]. The allele-specific real-time PCR utilized in the current enrolled clinical trials is an assay commercialized by DxS Ltd., which further guaranteed the high specificity [39]. In sum, to precisely direct personalized CRC treatment, we strongly recommend such highly specific methods or other methods with high specificity.

Other aspects to evaluate a PCR-based assay include throughput, contamination and convenience. The combination of PCR amplification and real-time PCR in these two types of PCR-based methods, PCR clamping-melting curve analysis and allele-specific real-time PCR, allows high-throughput and closed-tube test for detecting DNA mutations without cumbersome post-PCR methods. In addition, real-time monitoring template amplification significantly improved interpretation of PCR results [40]. At present, both methods have been successfully applied to search for different gene mutations in various tumor samples. For example, KRAS mutations in Bile [41], EGFR mutations in NSCLC [42], BRAF mutations in CRC [35]. The DxS also provides validated biomarker kit for EGFR, RAF, BCR-ABL and other genes that show a correlation between patient mutation status and drug response [39].

## Conclusion

In CRC anti-EGFR treatment, PCR-based assays with high sensitivity and specificity enable effective exclusion of patients with mutant KRAS, as well as reduce drug side effects and increase PFS and mOS in the wild-type KRAS patients. Simultaneously, such methods allow accurate identification of KRAS mutant patients so we can genuinely narrow the patient subgroup with KRAS mutations and investigate more effective treatment options for these patients. Therefore, an accurate standard reference test is urgently required to optimize mCRC-targeted therapy. Although the current PCR-based methods have performed much better than before, their sensitivity and specificity cannot be further improved due to the limited resolution of the analog signal and the unavoidable background molecules. This limitation considerably reduces the value of certain important clinical samples like stool and blood where target DNA only represents a small fraction of the total DNA. For example, tumor-derived DNA in the blood of patents with lower stage tumors is 0.01–0.12% [43]. The lately developed digital PCR (dPCR) method possesses the highest potential to improve detection accuracy in terms of both sensitivity and specificity. In dPCR, templates in a sample are compartmentalized into many minute individual reactions, each containing at most a single template. Such compartmentalization effectively decreases the noise and increases the amplification specificity of low-level templates [44], [45]. The reported sensitivity of dPCR reached 0.0005% [44]. More convenient than real-time PCR, dPCR doesn’t require the establishment of standard curve, and in terms of throughput the 96-well version dPCR has been developed [44], [45]. This promising method will allow us to substantially understand tumor heterogeneity and improve targeted cancer therapy [46], [47]. In conclusion, this research provides a much broader vision for the entire cancer therapy field.

## Supporting Information

Checklist S1 **PRISMA checklist.**
(DOC)
